# Evolution of the Surface Wettability of Vertically Oriented Multilayer Graphene Sheets Deposited by Plasma Technology

**DOI:** 10.3390/nano14121023

**Published:** 2024-06-13

**Authors:** Domen Paul, Rok Zaplotnik, Gregor Primc, Alenka Vesel, Miran Mozetič

**Affiliations:** Jožef Stefan Institute, Department of Surface Engineering, Teslova ulica 30, 1000 Ljubljana, Slovenia; rok.zaplotnik@ijs.si (R.Z.); miran.mozetic@ijs.si (M.M.)

**Keywords:** plasma, carbon, nanowalls, graphene, oxygen, cobalt, chemical vapor deposition, wettability

## Abstract

Carbon deposits consisting of vertically oriented multilayer graphene sheets on metallic foils represent an interesting alternative to activated carbon in electrical and electrochemical devices such as super-capacitors because of the superior electrical conductivity of graphene and huge surface–mass ratio. The graphene sheets were deposited on cobalt foils by plasma-enhanced chemical vapor deposition using propane as the carbon precursor. Plasma was sustained by an inductively coupled radiofrequency discharge in the H mode at a power of 500 W and a propane pressure of 17 Pa. The precursor effectively dissociated in plasma conditions and enabled the growth of porous films consisting of multilayer graphene sheets. The deposition rate varied with time and peaked at 100 nm/s. The evolution of surface wettability was determined by the sessile drop method. The untreated substrates were moderately hydrophobic at a water contact angle of about 110°. The contact angle dropped to about 50° after plasma treatment for less than a second and increased monotonously thereafter. The maximal contact angle of 130° appeared at a treatment time of about 30 s. Thereafter, it slowly decreased, with a prolonged deposition time. The evolution of the wettability was explained by surface composition and morphology. A brief treatment with oxygen plasma enabled a super-hydrophilic surface finish of the films consisting of multilayer graphene sheets.

## 1. Introduction

Plasma technologies are widely used for synthesizing nanomaterials [1] as well as for the preparation of substrates for the optimal adhesion of the nanomaterials on various substrates [2,3]. Plasmas sustained at both atmospheric and low pressure are useful for synthesizing and depositing nanomaterials. A large frequency of collisions governs atmospheric pressure plasmas, so the formation of clusters of condensable radicals is likely to occur, and the clusters tend to aggregate in the gas phase [4,5]. Appropriately designed experimental setups suppress the formation of aggregates in the gas phase, so the technology also enables the deposition of thin films with desired properties by plasma polymerization [6,7]. On the other hand, low-pressure plasmas often enable the deposition of uniform coatings free from aggregates, so they are widely used in industry [8,9]. Low-pressure plasma also enables the deposition of nanomaterials by the self-assembling condensable radicals on the substrate surface [10].

Carbon nanomaterials formed on substrates by self-assembling condensable radicals [11] are of particular interest. The method is known as Plasma-Enhanced Chemical Vapor Deposition (PECVD) and enables the deposition of various carbon-containing coatings from polymer-like films [12] to diamond nanoparticles [13]. The structure of the deposited carbon films depends on the plasma parameters, which in turn depend on the discharge parameters. Low specific discharge power (power per volume) and substrate temperature facilitate the deposition of polymer-like coatings [14], while powerful plasmas are useful for the deposition of diamond films. Various carbon nanomaterials of different compositions, structures, and morphological shapes grow between these two extremes, including fullerenes, nanotubes, nanowalls, and graphene sheets.

Graphene and similar materials (often referred to as doped graphene) are among the most promising electrodes for super-capacitors because of their low weight, chemical stability, and large surface–mass ratio [15,16]. Although their energy capacity is inferior to batteries, super-capacitors have other attractive properties as they enable high-speed energy delivery, faster charge–discharge speeds, longer lifetimes, and reusability [17]. Classical super-capacitors are often made from activated carbon [18] and often produced from renewable sources [19]. Electrodes made from activated carbon enable a large surface–mass ratio and thus a specific capacitance [20], but the rather inadequate electrical conductivity of activated carbon limits power density and prevents fast charging–discharging.

An alternative to activated carbon is depositing vertically oriented graphene-like materials onto a metallic foil by PECVD. Furthermore, graphene is known for its excellent electrical conductivity. The first report on the deposition of vertically oriented multilayer graphene sheets by PECVD was provided by Wu et al. [21], with a later report provided by the Hori group [22]. Hydrocarbon gas is often mixed with argon and hydrogen, and high-quality carbon nanowalls grow on the surface of a substrate at elevated temperatures [23]. The carbon precursor is usually methane [24], but higher hydrocarbons have only been used for the synthesis of graphene flakes at elevated pressure [25]. The wettability of vertically oriented graphene flakes has rarely been reported [26], although it may influence the ability to assemble a top-quality super-capacitor [27]. The wettability of graphene flakes is inadequate for specific applications but could be increased by a brief treatment with oxygen plasma [28].

The present paper describes the wettability of vertically oriented multilayer graphene sheets deposited on a metallic substrate by PECVD. The carbon precursor was propane, which enables a faster deposition rate than methane (let alone a mixture of methane with hydrogen and a noble gas) at low-pressure conditions.

## 2. Materials and Methods

**Figure 1:**
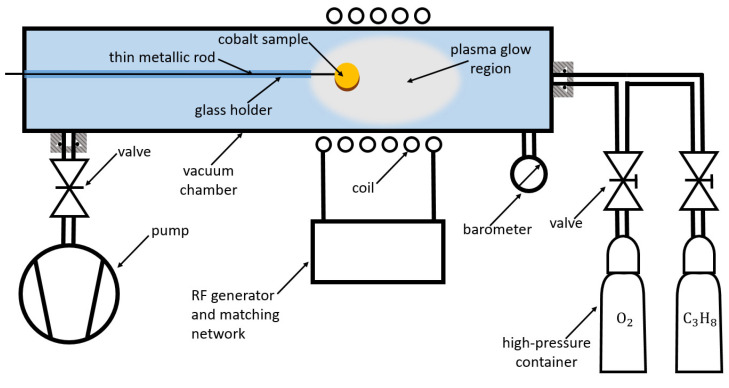
Schematic of the experimental setup used to synthesize multilayer graphene sheets on the cobalt disk.

Vertically oriented multilayer graphene sheets were deposited on well-oxidized cobalt samples employing a PECVD method. A schematic of the experimental setup is shown in Figure 1. The discharge tube, with an inner diameter of 3.6 cm, was made from borosilicate glass. The tube was pumped on one side with a two-stage rotary pump with a nominal pumping speed of 80 m^3^/h. On the other side, commercially available gases were leaked into the tube through a manually adjustable needle valve. We used either oxygen with a purity of 99.99%+ or propane with a purity of about 99%. A low-pressure discharge was sustained with a radio frequency (RF) generator with a frequency of 13.56 MHz and with a forward power of 500 W. The generator was hooked to a computer and controlled remotely through proprietary software. The generator was connected inductively to a water-cooled copper coil via a matching network. The plasma of high luminosity (H-mode) was inside the coil, in a range of pressures up to about 50 Pa. The experiments were performed at the oxygen or propane gas pressure with the position of the barometer around 17 Pa. A 0.05 mm thick cobalt (99.8% purity) disk with a 3 mm diameter was spot-welded to a thin metallic rod, which was fixed onto the flange far away from the luminous plasma, as shown in Figure 1. Therefore, the cobalt disc was exposed to radicals created in the plasma sustained in the H-mode. The dense plasma enabled substantial dissociation of oxygen or the propane precursor and the partial ionization of atoms and propane molecular fragments. The dissociation fraction of oxygen was determined with a calibrated catalytic probe, as explained in detail elsewhere [29].

The fragments formed in the propane plasma condensed on the cobalt disc, forming carbon coatings. Scanning electron microscopy (SEM) monitored the carbon deposits’ morphology. We used Schottky field emission SEM with a Thermo Fisher Verios 4G HP monochromator (Thermo Fisher Scientific, Waltham, MA, USA).

Additional chemical analysis was performed using X-ray photoelectron spectroscopy (XPS). The model used was manufactured by TFA XPS Physical Electronics in Germany (Physical Electronics, Feldkirchen, Germany).

The carbon deposits are likely to be hydrophobic. The hydrophilicity of the as-deposited carbon films was obtained by brief exposure to oxygen plasma sustained in the same reactor (Figure 1).

Measurements employing atomic force microscopy (AFM) were performed with Solver SPM produced by NT-MDT (Moscow, Russia). Measurements were performed in non-contact mode with a recording frequency of 2 Hz and the resolution of the images was set to 256 × 256 pixels.

The wettability was measured by the sessile drop method. We used a KRUSS Drop Analyzer D5A100E (KRUSS Scientific, Hamburg, Germany) to deposit the water droplets on our samples and the device’s built-in camera to analyze the drops on the sample’s surface. The water droplets were all of uniform volume (2.67 μL), and 5 repetitions of every measurement were performed.

## 3. Results and Discussion

Systematic research on the wettability of the cobalt samples, either activated by exposure to oxygen plasma or coated with carbon deposits, was performed. A cobalt substrate was connected to the thin metallic rod and exposed to plasma in the system, as shown in Figure 1. After the treatment, the system was vented, and the cobalt disc was cut from the metallic rod and characterized for wettability. SEM also characterized selected samples. Numerous cobalt discs were prepared. The coating with nanocarbon was accomplished using propane plasma, and the deposition time to obtain the carbon films varied between 0.1 and 300 s. The activation of the carbon deposits was achieved via treatment with diffusing oxygen plasma sustained in the discharge tube outside the RF coil (Figure 1).

### 3.1. Wettability of Cobalt Samples

**Figure 2:**
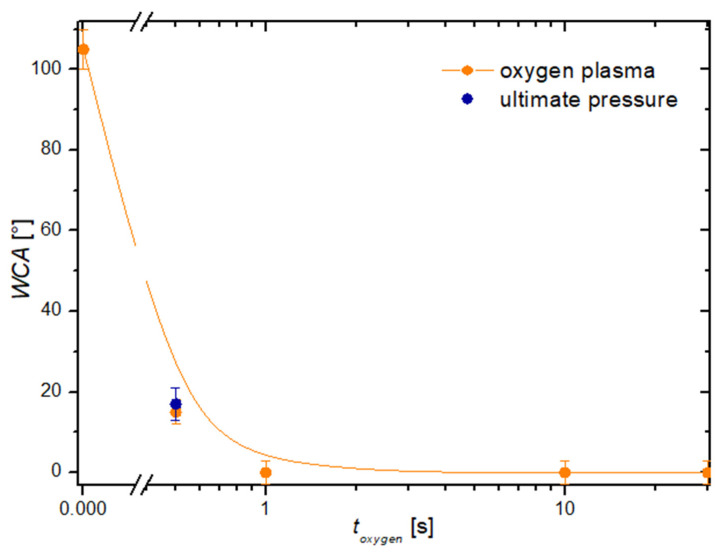
The water contact angle versus the treatment time of cobalt samples treated with oxygen plasma.

**Figure 3:**
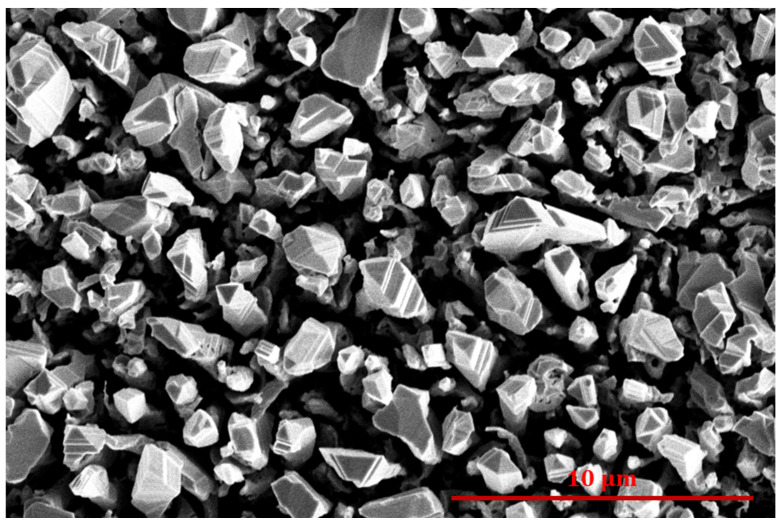
SEM image of a cobalt disk treated with oxygen plasma for 10 s.

The water contact angle (WCA) for the pristine cobalt sample was about 110°. This value is typical for metallic samples stored at ambient conditions. Specifically, metals are likely to adsorb organic vapor, which is present in the ambient air in minute quantities. The adsorbed organic impurities govern the wettability of the metallic samples [30]. The cobalt substrates were treated with a plasma sustained in an oxidizing atmosphere. We used both plasma sustained at the ultimate pressure and plasma sustained in oxygen, with the ultimate pressure of 1 Pa achieved simply by pumping out our experimental systems, with no added gases. Figure 2 reveals the wettability of cobalt substrates treated with such plasmas. The WCA decreased significantly even after exposure of the cobalt substrate to plasma sustained at the ultimate pressure. Specifically, the WCA dropped to about 30° even after the treatment at ultimate pressure for 0.5 s. Even better wettability was obtained by treating the cobalt substrates in oxygen plasma. The evolution of the water contact angle versus the oxygen plasma treatment time is shown in Figure 2. A treatment with oxygen plasma for 0.5 s caused a WCA of about 15°, but longer treatment times resulted in the super-hydrophilic surface finish because the WCA became immeasurably low. In fact, a water droplet spread on a large surface after the treatment of the cobalt substrates for more than 1 s. An SEM image of the sample treated in oxygen plasma for 10 s is shown in Figure 3. One can observe a very rich morphology, which is due to the growth of the oxide film. Furthermore, the treatment of metallic foils with oxygen plasma often results in nanostructured oxide films [31,32]. Such a rich morphology is not appropriate for studying the wettability of samples coated with nanocarbon, so these experiments were performed using untreated cobalt samples.

### 3.2. Wettability of Nanocarbon Deposits on Cobalt Substrates

**Figure 4:**
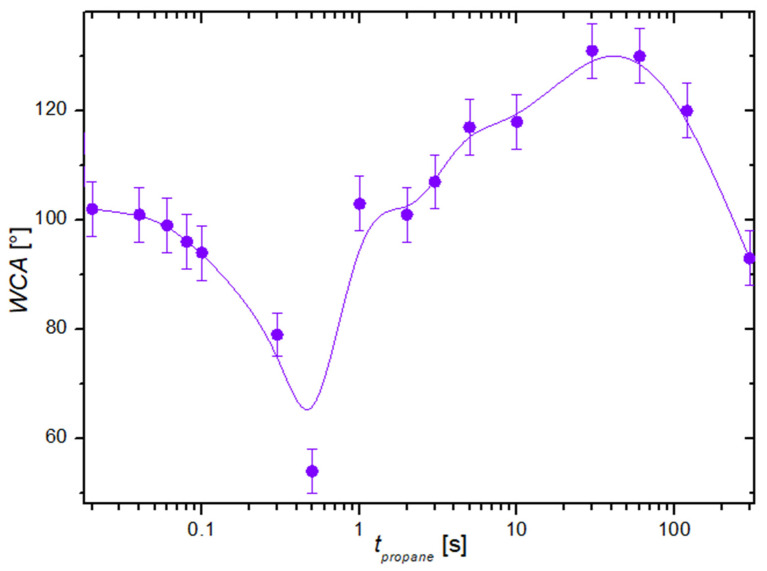
The water contact angle versus the treatment time of cobalt samples treated with propane plasma.

**Figure 5:**
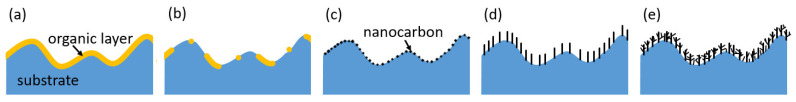
Illustration of the surface finish governing the wettability of cobalt samples upon treatment with propane plasma. (**a**) The untreated samples are covered with a very thin film of organic vapors adsorbed when storing substrates in the air; (**b**) treatment in propane plasma for 0.1 s causes partial removal of the adsorbed organic impurities; (**c**) when the substrate is almost free from either organic impurities or graphene flakes (about 0.5 s), the WCA assumes the minimal value; (**d**) treatment for about 30 s enables deposition of vertically oriented graphene flakes, so the WCA peaks; (**e**) prolonged treatment causes densification of the graphene flakes and thus only moderate hydrophobicity.

Figure 4 shows the evolution of the water contact angle versus the deposition time when using propane plasma. Even a brief treatment with a plasma sustained in the propane gas causes a significant decrease in the WCA and, thus, increased wettability. As illustrated in Figure 5b, the effect is explained by the desorption of the organic impurities. The WCA drops to about 50° after a treatment time of about 0.5 s. This treatment time probably represents the balance between two opposite effects: 1—the removal of the adsorbed organic impurities increases with increasing plasma treatment time, and 2—the coverage of the substrates with the nanocarbon increases with increasing treatment time in propane plasma.

After a second of propane plasma treatment, the nucleation of nanocarbon becomes large enough to cover practically the entire cobalt surface, as shown in Figure 5c. Thereafter, the WCA increases with increasing treatment time because the deposits, in the form of vertically oriented graphene sheets, keep growing, so the surface morphology changes. The wettability depends on the roughness of the solid materials on the sub-micrometer scale [33,34]. As a rule of thumb, the WCA increases with increasing surface roughness as long as the materials are not highly hydrophilic [35]. In fact, the super-hydrophobic surface finish is a result of a hydrophobic coating with a rich morphology [26,36,37]. The increase in the WCA in the range of deposition times between about 0.5 and 50 s in Figure 4 is thus explained by the increasing roughness of the surface, which is a result of the deposition of vertically oriented multilayer graphene sheets, as illustrated in Figure 5d.

Interestingly enough, the WCA decreases after a prolonged deposition time. This effect is explained by the branching of the graphene flakes, which decreases the roughness of the nanocarbon at prolonged deposition times.

**Figure 6:**
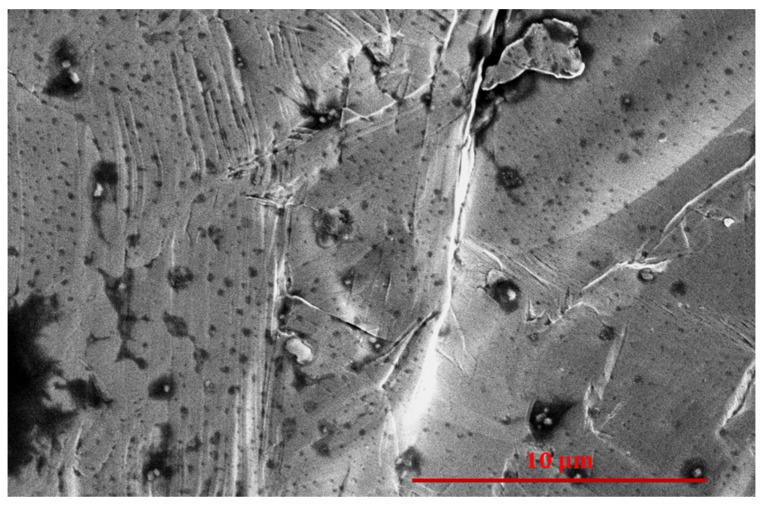
SEM image of an untreated cobalt sample.

**Figure 7:**
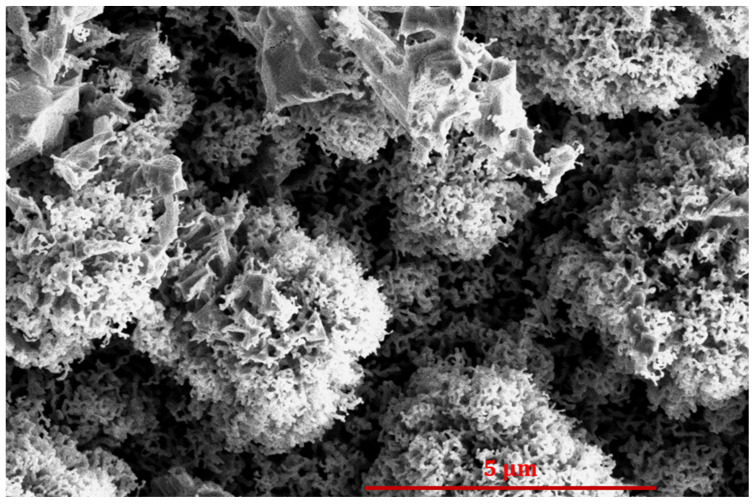
SEM image of a cobalt sample treated with propane plasma for 1 s.

The illustrations in Figure 5 are supported by SEM images. Figure 6 shows an SEM image of an untreated cobalt disk. There are some holes and voids on the surface, but the material is rather flat, without significant structuring on a sub-micrometer scale. As mentioned earlier, the untreated cobalt samples are also covered with a layer of organic impurities, so the WCA (Figure 4) is rather large, which is typical for organic materials. The surface morphology, as deduced by SEM images, remains fairly unchanged until a propane plasma treatment time of about 1 s. Figure 7 shows an SEM image of such a sample. One can still observe grains of metal, but the majority of the surface is already covered with nanocarbons of irregular morphology. The wettability of the sample treated for 1 s is still moderately low because the surface roughness on the sub-micrometer scale is rather low, as revealed in Figure 7.

**Figure 8:**
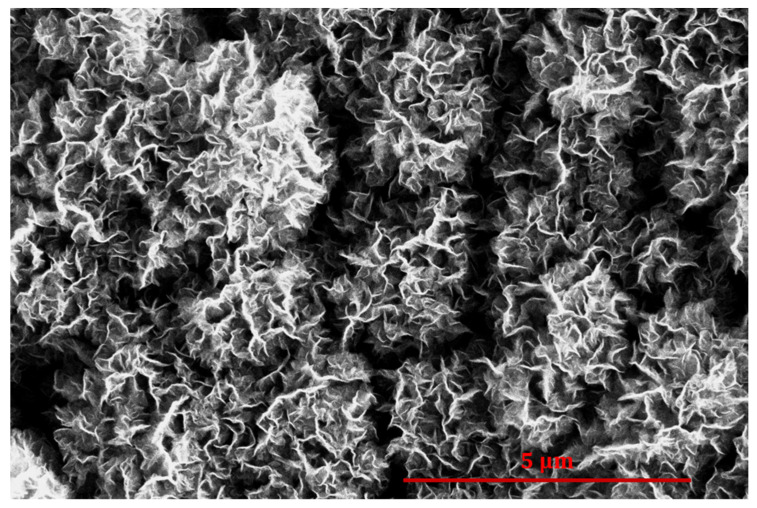
SEM image of a cobalt sample treated with propane plasma for 30 s.

Increasing the treatment time causes the growth of almost-vertically oriented graphene flakes, as shown in Figure 8. This image was taken after treating a sample in propane plasma for 30 s. One can observe vertically oriented nanocarbon, with the distance between two neighboring multilayer graphene walls in the order of 0.1 μm. Such a rich morphology enables very large hydrophobicity. Figure 4 shows the WCA as large as about 130° for the sample treated with propane plasma for 30 s. This treatment time, therefore, enables optimal hydrophobicity, as long as this is the requirement. Prolonged treatment, however, causes a slight decrease in the WCA, as revealed in Figure 4. This is explained by the loss of the rich morphology shown in Figure 8.

**Figure 9:**
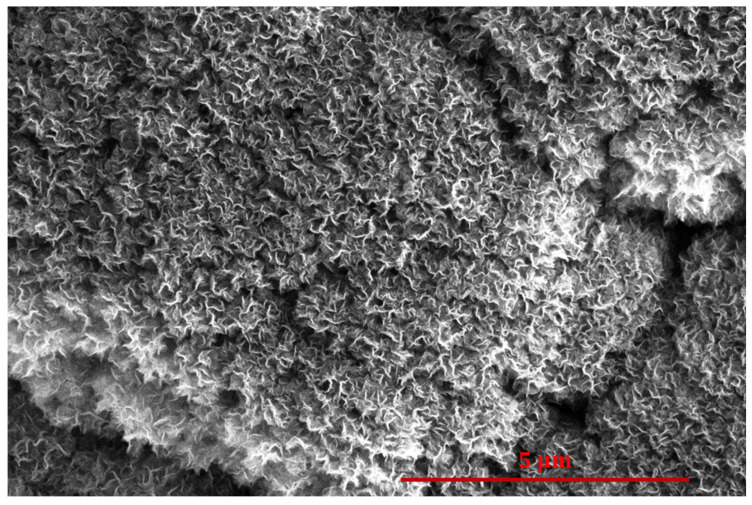
SEM image of a cobalt sample treated with propane plasma for 300 s.

Figure 9 represents an SEM image of a sample treated for 300 s. The morphology is much different from that shown in Figure 8 because the graphene structures are now much denser. Such a dense distribution of nanocarbon is explained by branching, i.e., by the growth of small nanowalls between the originally vertical structures typical for moderate treatment times (Figure 8). As a result, the gaps between neighboring nanowalls are much narrower in Figure 9 than in Figure 8. The morphology shown in Figure 9 still enables a hydrophobic character of coating, but the very high hydrophobicity cannot be obtained with such densely packed, randomly oriented nanocarbon structures.

**Figure 10:**
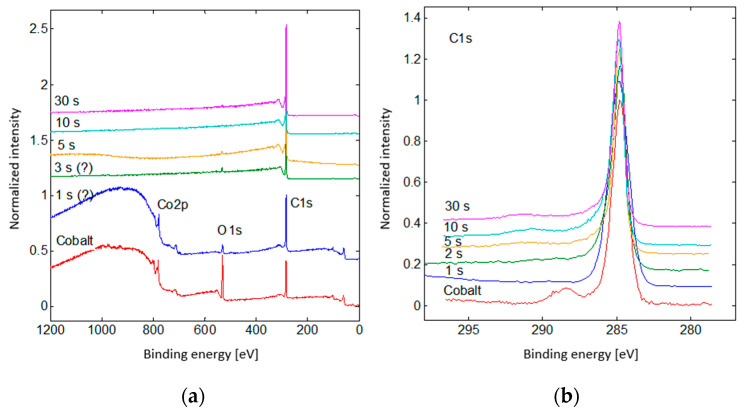
Spectra of the binding energies (XPS) (**a**) and high resolution spectra of carbon (**b**), as measured with the XPS method.

**Figure 11:**
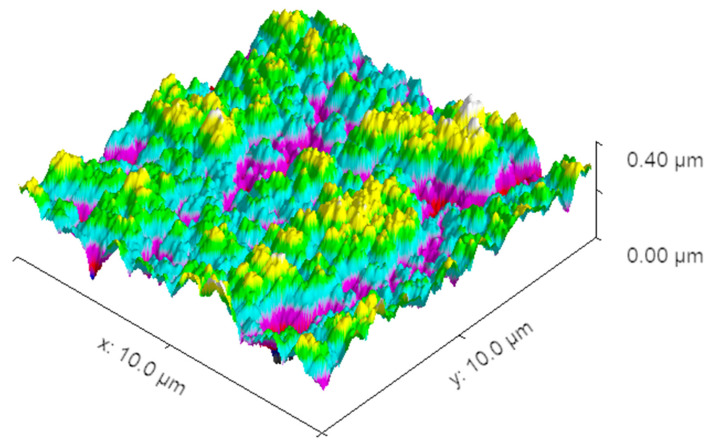
AFM image of a cobalt sample after 30 s deposition time of CNW.

XPS was utilized to study the chemical composition of the surface of our samples. The results are the spectra shown in Figure 10a. Additionally, the high-resolution spectra in the region of 290–295 eV are shown in Figure 10b. The amount of carbon, oxygen, and cobalt for each of those spectra is shown in Table 1. Additionally, AFM measurements were performed on a cobalt sample with deposited CNW (Figure 11). As expected, the results are fairly similar to those in Figure 8.

### 3.3. Wettability of Samples with Carbon Deposits Activated with Oxygen Plasma

The hydrophobic character of the deposits may be useful in particular applications, but in many other applications the opposite effect is desired, i.e., good wettability and the ability to be soaked with liquids. These applications include electrochemical devices such as super-capacitors. In order to change the surface properties of as-deposited nanocarbon coatings, we briefly treated them with oxygen plasma. After the deposition of the nanocarbon, we moved a sample inside the reactor to outside the position of the powerful plasma in the H mode (Figure 1) and sustained the plasma in oxygen. The plasma treatment time varied between 0.1 and 1 s. All samples became super hydrophilic after such a brief oxygen plasma treatment because the WCA was well below the detection limit. In fact, the water was soaked by the nanocarbon structures in less than a second after the deposition of the water droplet. The brief treatment of carbon nanostructures with oxygen plasma, therefore, enables a change from a highly hydrophobic to a super hydrophilic surface finish. The functionalization of nanocarbon with oxygen explains the change in the surface wettability of carbon deposits. It is well known that oxygen-containing functional groups are hydrophilic. Furthermore, the rich morphology enables the super hydrophilic surface finish because capillary forces drag any polar liquid into the gaps.

The super hydrophilic surface finish goes against the rules of thermodynamics, so keeping it for a long time is impossible. The spontaneous loss of such a surface finish has been observed for all materials, and the effect is usually called hydrophobic recovery [38]. While the exact mechanisms of hydrophobic recovery are yet to be elaborated, they are usually explained by the loss of polar surface functional groups, the reorientation of the functional groups into the interior of the substrate, or simply the covering of the materials with organic impurities. Whatever the mechanism, it is useful to study hydrophobic recovery and estimate the loss of this effect.

**Figure 12:**
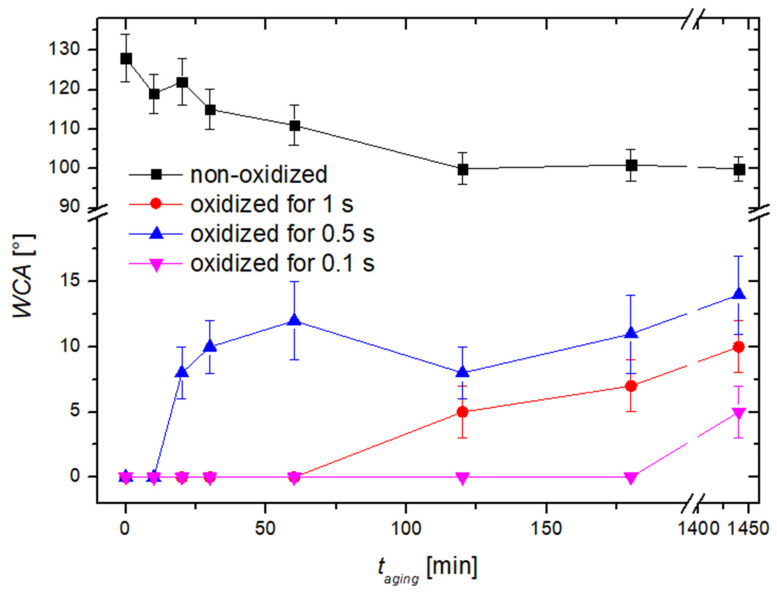
The WCA of aging samples, coated with multilayer graphene flakes using propane plasma for 30 s, and then treated with oxygen plasma for 0.1, 0.5, or 1 s.

Hydrophobic recovery was studied for samples treated with propane plasma for 30 s. Figure 4 reveals the highly hydrophobic character of such samples. These samples were treated with oxygen plasma for 0.1, 0.5, and 1 s and stored at ambient conditions. The hydrophobic recovery was determined over three days. The results are shown in Figure 12. As expected, the super hydrophilic surface finish is lost after storage at ambient conditions. Interestingly enough, however, is the fact that a very low WCA of about 10° is preserved even after an hour of aging. The results shown in Figure 12, therefore, indicate a rather slow hydrophobic recovery. Moreover, the hydrophobic recovery of most super hydrophilic carbon materials like polymers is much faster [39,40]. As has already been mentioned, the exact mechanisms of hydrophobic recovery are yet to be elaborated, especially for highly hydrophilic nanocarbon materials. The hydrophobic recovery of polymers is often attributed to the reorientation of the polar function groups and even the diffusion of oxygen into the subsurface structures. Multilayer vertically oriented graphene sheets are among the very compact materials where diffusion is unlikely to occur at room temperature. The reorientation or loss of polar surface functional groups is thus highly improbable for the materials deposited by our method. As mentioned earlier, many solid materials are likely to bind organic impurities from ambient air. This is the reason for the hydrophobic character of untreated cobalt samples. The hydrophobic recovery of metals is fast, so the results presented in Figure 10 indicate that the adsorption of organic impurities proceeds rather slowly on activated multilayer graphene sheets.

**Figure 13:**
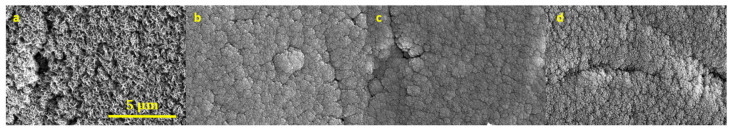
The SEM images of CNW (30 s of PECVD on a cobalt surface) after (**a**) 0, (**b**) 0.1, (**c**) 0.5, and (**d**) 1 s of treatment in the glowing region of H-mode oxygen plasma. The scale is the same in all the images.

The uppermost curve in Figure 12 is of a sample without subsequent treatment with oxygen plasma. The WCA slowly decreases for this sample. The decrease in the WCA could be a result of the adsorption of organic impurities upon storage at ambient conditions. Additional SEM images of the oxidized CNW samples are shown in Figure 13. There were no observable differences to the surface morphology after 0.1 (Figure 13b) and 0.5 (Figure 13c) s of oxygen plasma treatment when compared to the untreated CNW (Figure 13a). However, some etching occurred after treating the CNW for 1 s with oxygen plasma (Figure 13d), possibly linking the etching effect to the hydrophobic recovery in Figure 12.

## 4. Conclusions

We studied the wettability of nanocarbon materials synthesized by the PECVD method using propane as the carbon precursor. The first effect of the plasma treatment is the desorption of organic impurities from the cobalt substrates, which causes a gradual decrease in the water contact angle. Vertically oriented multilayer graphene sheets, however, exhibit very high hydrophobicity, since the WCA is about 130°. Such a high WCA effectively prevents the interaction of polar liquids with nanocarbon materials, but facilitates interaction with non-polar materials such as organic impurities in the water, so these materials may be useful for water purification, especially for the removal of traces of oil from contaminated waters. A highly hydrophobic character limits the ability of as-deposited nanocarbon materials to be impregnated with various liquids, including electrolytes. Fast impregnation with such liquids is achievable by a brief treatment of the nanocarbon materials with oxygen plasma. Even 0.1 s of oxygen plasma treatment causes a super hydrophilic surface finish of nanocarbon, and thus rapid impregnation with any liquid, including water, which is among the liquids with the highest polarity. The super hydrophilic surface finish is lost within an hour or so, so it persists long enough to enable further treatments such as impregnation with liquids.

## Figures and Tables

**Table 1:** Composition of the surface of cobalt samples with deposited CNW for different deposition times, as measured with the XPS method.

PECVD Time [s]	C	O	Co
0	56.4	32.3	11.3
1	87.7	6.2	6.1
3	96.5	3.5	0
5	98.3	1.7	0
10	99.2	0.8	0
30	99.2	0.8	0

## Data Availability

The data that support the findings of this study are available upon reasonable request from the authors.
